# Genetic basis of arsenite and cadmium tolerance in *Saccharomyces cerevisiae*

**DOI:** 10.1186/1471-2164-10-105

**Published:** 2009-03-12

**Authors:** Michael Thorsen, Gabriel G Perrone, Erik Kristiansson, Mathew Traini, Tian Ye, Ian W Dawes, Olle Nerman, Markus J Tamás

**Affiliations:** 1 Department of Cell and Molecular Biology/Microbiology, University of Gothenburg, S-405 30 Gothenburg, Sweden; 2 Ramaciotti Centre for Gene Function Analysis, School of Biotechnology and Biomolecular Sciences, University of New South Wales, Sydney, NSW 2052, Australia; 3 Department of Mathematical Statistics, Chalmers University of Technology/University of Gothenburg, S-412 96 Gothenburg, Sweden; 4 Department of Biology, University of Copenhagen, Copenhagen, Denmark

## Abstract

**Background:**

Arsenic and cadmium are widely distributed in nature and pose serious threats to the environment and human health. Exposure to these nonessential toxic metals may result in a variety of human diseases including cancer. However, arsenic and cadmium toxicity targets and the cellular systems contributing to tolerance acquisition are not fully known.

**Results:**

To gain insight into metal action and cellular tolerance mechanisms, we carried out genome-wide screening of the *Saccharomyces cerevisiae *haploid and homozygous diploid deletion mutant collections and scored for reduced growth in the presence of arsenite or cadmium. Processes found to be required for tolerance to both metals included sulphur and glutathione biosynthesis, environmental sensing, mRNA synthesis and transcription, and vacuolar/endosomal transport and sorting. We also identified metal-specific defence processes. Arsenite-specific defence functions were related to cell cycle regulation, lipid and fatty acid metabolism, mitochondrial biogenesis, and the cytoskeleton whereas cadmium-specific defence functions were mainly related to sugar/carbohydrate metabolism, and metal-ion homeostasis and transport. Molecular evidence indicated that the cytoskeleton is targeted by arsenite and that phosphorylation of the Snf1p kinase is required for cadmium tolerance.

**Conclusion:**

This study has pin-pointed core functions that protect cells from arsenite and cadmium toxicity. It also emphasizes the existence of both common and specific defence systems. Since many of the yeast genes that confer tolerance to these agents have homologues in humans, similar biological processes may act in yeast and humans to prevent metal toxicity and carcinogenesis.

## Background

The presence of nonessential metals like arsenic (As) and cadmium (Cd) in the environment is prevalent. Since these metals are highly toxic, they pose a considerable threat to nature and to human health. The main routes of poisoning are through occupational exposure or through ingestion of contaminated food and water. Pollution of soils with toxic agents is a common global problem, and contamination of drinking water by arsenic is a major health concern because of the large number of contaminated sites and people at risk. These metals are implicated in a broad spectrum of degenerative conditions in humans, including neurotoxicity, nephrotoxicity, genotoxicity and carcinogenesis. For example, chronic As exposure induces cardiovascular diseases, neurological disorders and liver injury, and is associated with cancers of the skin, bladder, liver and lung. Cd is considered genotoxic and carcinogenic for lung, kidney and prostate [1-4]. The International Agency for Research on Cancer (IARC) classifies both metals as group I carcinogens [5].

The toxicity mechanisms of As and Cd are not fully understood at the molecular level. In general, they may act by targeting signalling or regulatory proteins that control cell proliferation, differentiation and cell cycle regulation. Although the mode of action of each metal is likely to involve unique features, some toxicity mechanisms may be shared. Similarly, cells may mount both common and metal-specific responses to counteract toxicity [2,6-8]. A common property of As and Cd is their high reactivity with sulphhydryl groups. Hence, they can bind to and affect the activity of many proteins. In addition, these metals are known to generate oxidative stress in cells and their toxicity has partly been attributed to their capability to induce formation of reactive oxygen species (ROS). The damage caused by ROS to lipids, proteins and DNA are likely to contribute to As and Cd toxicity [7,9]. Nevertheless, neither the exact details of metal-induced ROS generation nor the full set of toxicity targets is known.

Drugs containing arsenicals are currently used in medical treatment. The arsenic-containing drug melarsoprol^® ^is used to treat diseases caused by the protozoan parasite *Trypanosoma *[10,11], whereas arsenic trioxide (Trisenox^®^) is used as a treatment for acute promyelocytic leukaemia and it may also be employed for other haematological and solid cancers [12]. However, the emergence of resistance threatens the efficacy of medical treatment [13] and hence, there is an increasing demand to identify tolerance mechanisms. Similarly, the prospect of using plants to clean up polluted soils has recently attracted considerable attention. Nevertheless, to develop phytoremediation into a useful approach requires a detailed understanding of the genetics and molecular basis of detoxification and tolerance acquisition [14,15].

Tolerance and detoxification mechanisms often involve extrusion of the toxic ions from the cell, sequestration within internal organelles, chelation by metal-binding proteins, and reduction of uptake. Common to these systems is that they reduce the cellular content of the toxic agent, although their molecular basis may differ between metals and also between organisms [16-19]. Furthermore, it is clear that not only the detoxification systems themselves, but also the proteins that regulate their expression, localization and/or activity will contribute to cellular metal tolerance.

The aim of this work was to provide a global view of the genetic basis of As and Cd toxicity and detoxification by identifying the molecular/cellular targets of their action and to reveal tolerance acquisition mechanisms. Unveiling metal toxicity and tolerance mechanisms in yeast may prove of value for identifying similar mechanisms in higher eukaryotes.

## Results and discussion

### Identification of arsenite and cadmium sensitive yeast mutants

**Figure 1:**
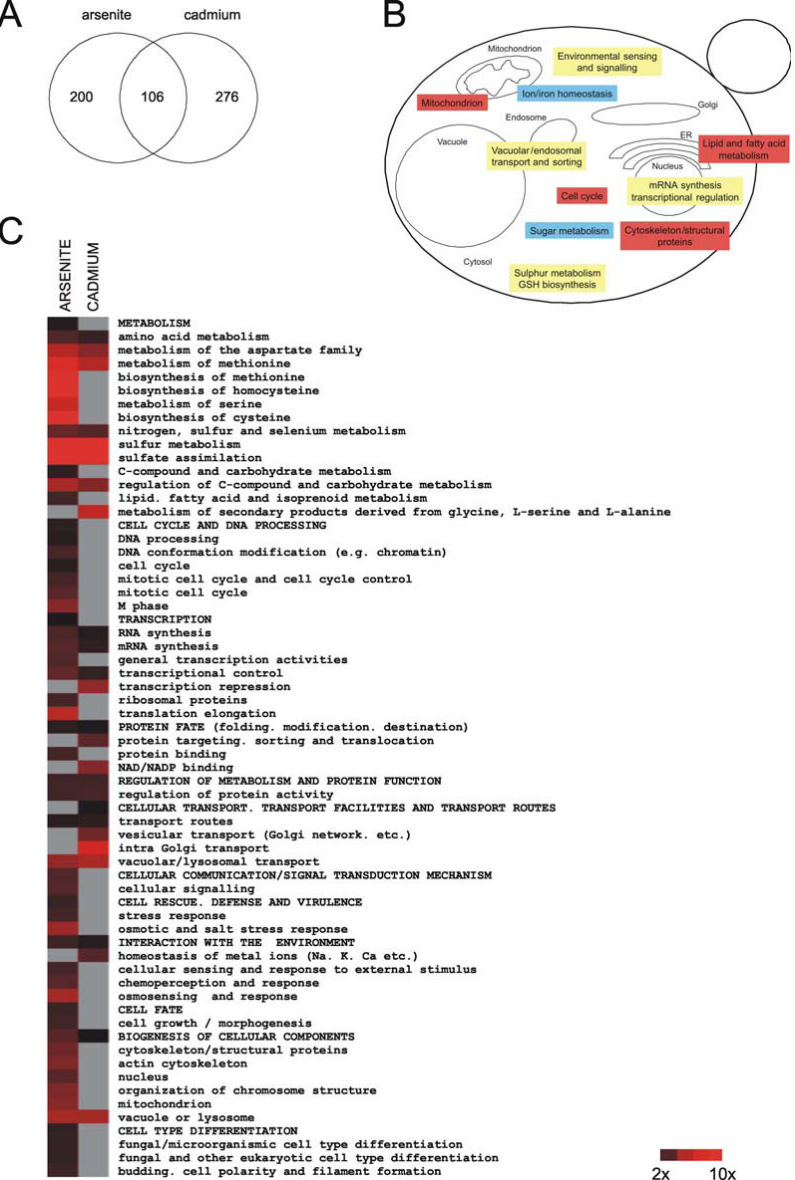
**Analysis of yeast genes conferring arsenite and cadmium tolerance**. (**A**) Venn diagram visualizing unique and common As and Cd sensitive yeast mutants identified in this work. (**B**) Model showing selected functional categories (according to FunCats (MIPS)) that are affected by As and Cd. Yellow: function required for both As and Cd tolerance; Red: function required for As tolerance; Blue: function required for Cd tolerance. (**C**) Heat map depicting all functional categories in our data set that were significantly (*p*-value < 0.005) enriched for As and Cd sensitive mutants. Parent terms are in capitals, child terms in lower case letters. The colour indicates the fold enrichment of genes within individual categories. Gray boxes indicate a category that did not pass the significance (*p*-value < 0.005) cut-off.

We individually exposed ~4700 haploid *Saccharomyces cerevisiae *gene deletion mutants (the entire set of nonessential genes in this yeast) to arsenite and cadmium. The screen was limited to the trivalent form of arsenic (arsenite) since arsenite is more toxic than arsenate and is the form that is principally responsible for the biological effects of arsenic in medical therapy. Cells were exposed to increasing concentrations of each metal (3 doses) on solid medium and their growth was monitored by digital imaging and computational analysis as described in the Methods section. Deletion strains were considered sensitive if they showed a significant growth reduction in the presence of metal compared to its growth in the absence of metal. We also screened the homozygous diploid strain collection to ensure that phenotypes manifested only in haploid or diploid strain backgrounds were identified. In this case, homozygous deletion strains that exhibited reduced growth relative to the wild-type in liquid medium containing metal were identified as sensitive. Sensitivity of these strains was confirmed by re-screening. The combined set of deletion strains from the two screens (haploid and homozygous diploid) comprised 306 arsenite-sensitive and 382 cadmium-sensitive mutants. 106 mutants (18%) were sensitive to both metals (Fig 1A). For a list of all As and Cd sensitive mutants, see Additional file 1.

### Cellular functions contributing to both arsenite and cadmium tolerance

To pin-point cellular functions that protect cells from metal toxicity, we searched for functional categories (according to FunCat, Munich Information Center for Protein Sequences (MIPS) [20]) that were significantly enriched (*p*-value < 0.005) in the sensitive gene set as compared to the whole genome. As was the case with individual genes (see above), this analysis revealed common as well as metal-specific tolerance functions (Fig 1). Prominent among those required for both As and Cd tolerance, were functions related to methionine and sulphur metabolism, mRNA synthesis and transcriptional regulation, environmental sensing, and vacuolar/endosomal transport and sorting. The *p*-values and fold enrichment factors of genes within individual functional categories are available in Fig 1C and Additional file 1.

#### Methionine and sulphur metabolism

Cells respond to As or Cd exposure by inducing expression of genes and enzymes in the sulphur assimilation and glutathione biosynthesis pathways. Moreover, cells also channel a large part of assimilated sulphur into biosynthesis of the low molecular weight thiol molecule glutathione [21-24]. Glutathione is important for metal tolerance since it may protect cells by: 1) metal chelation and sequestration; 2) protection against metal-induced oxidation since glutathione is considered the main redox buffer of the cell; and 3) binding to reactive sulphhydryl groups on proteins (glutathionylation) to shield them from irreversible metal binding and/or oxidative damage [25,26]. The fact that we identified several metal sensitive mutants having functions in the sulphur assimilation and glutathione biosynthesis pathways as well as transcriptional regulators controlling these pathways, underscores the importance of the sulphur/glutathione biosynthesis pathways for tolerance acquisition. Moreover, the *ycf1*Δ mutant lacking an ABC-type transporter responsible for sequestration of metal-glutathione conjugates into vacuoles [27-29] was also As and Cd sensitive. The enzymes in the sulphur assimilation/glutathione biosynthesis pathways are transcriptionally regulated by the transcription factors Yap1p and Met4p, and cells lacking any of these factors display As and Cd sensitivity [21,22]. Met4p is recruited to target promoters by the DNA-binding proteins Met31p, Met32p and Cbf1p forming the complexes Met4p-Met31p/Met32p and Met4p-Cbf1p. An additional protein, Met28p stabilizes the Met4p-containing complexes [30]. Our data confirms the importance of Yap1p for metal tolerance and suggests that different Met4p-complexes may be important during As and Cd exposure; mutants lacking *MET28 *and *CBF1 *were As-sensitive whereas cells lacking *MET32 *and *MET28 *were Cd-sensitive. Taken together, these findings are consistent with the central role of sulphur metabolism and glutathione biosynthesis for As and Cd tolerance.

#### mRNA synthesis and transcriptional regulation

As or Cd exposure results in altered expression of a large number of genes [21,22,31-33]. Accordingly, mutants defective in mRNA synthesis and transcriptional control were sensitized to both metals (Fig 1). Among the gene-specific regulators that confer metal tolerance was Yap1p (regulates expression of genes with functions in oxidative stress defence and sulphur/glutathione metabolism), Met28p, Cbf1p, Met32p (sulphur/glutathione metabolism) and Rpn4p (protein degradation). Transcriptional activators may stimulate gene-specific expression by recruiting the co-regulator complexes Mediator, SAGA and/or Swi/Snf as well as RNA polymerase II to promoters. Indeed, several metal-sensitive mutants lacked subunits of RNA polymerase II (*rpb4*Δ, *rpb9*Δ) or components of the Mediator (*pgd1*Δ, *srb8Δ*), SAGA (*gcn5*Δ, *ada2*Δ, *spt7*Δ), and Swi/Snf (*swi3*Δ, *snf2*Δ) co-regulator complexes. Although sensitivity of these mutants could be a result of a general impairment of transcriptional activity, both Yap1p and Met4p interact physically with components of these co-regulator complexes [34,35].

#### Interaction with the environment

Cells defective in functions related to environmental sensing and signalling were metal sensitive (Fig 1). The limited overlap between the As and Cd sensitive mutants within this category suggests that cells may use distinct sensing/signalling pathways in response to these metals. For instance, cells appear to use distinct MAP kinase pathways for sensing/responding to As and Cd since mutants defective in the cell integrity pathway (*slg1*Δ, *bck1*Δ, *slt2*Δ) were preferentially Cd sensitive whereas cells defective in the osmosensing HOG pathway (*ssk1*Δ, *ssk2*Δ, *pbs2*Δ, *hog1*Δ) were preferentially As sensitive. We note that cells lacking the HOG pathway components Ssk1p and Hog1p were also Cd sensitive. The As and Cd-sensitivity of the latter mutants may reflect different functions/targets of the corresponding proteins under the two stress conditions. Hog1p is phosphorylated in response to As and contributes to tolerance by at least two mechanisms; first, Hog1p restricts arsenite influx through the aquaglyceroporin Fps1p by phosphorylating this protein (deletion of *FPS1 *results in arsenite resistance) [36,37]. Secondly, Hog1p regulates the exit from As-induced G_1 _arrest [38]. Hog1p is also phosphorylated in response to Cd [39] but the target(s) and mechanism(s) through which Hog1p mediates Cd tolerance are unknown. Interestingly, Hog1p has a role in the cell integrity pathway and the cellular response to cell wall damage involves both the Hog1p and Slt2p/cell integrity MAP kinase pathways [40]. The fact that cells lacking *HOG1 *or components of the cell integrity pathway are Cd sensitive, suggests that the role of Hog1p under Cd exposure may be linked to cell wall damage.

#### Vacuolar/endosomal transport and sorting

**Figure 2:**
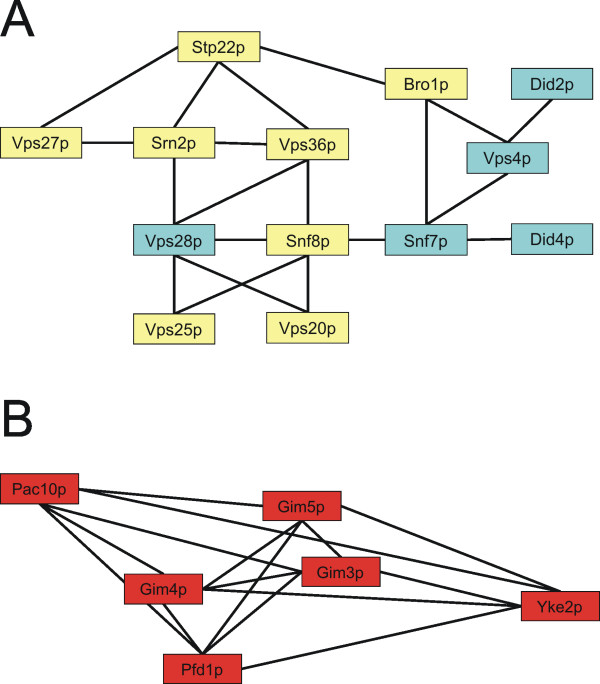
**Example of protein complexes affected by arsenite and cadmium**. (**A**) The ESCRT complex and (**B**) the GIM complex. Proteins of these complexes that confer tolerance to both metals are labelled in yellow, those that confer Cd tolerance are labelled in blue, and those that confer As tolerance are labelled in red. The lines indicate physical interactions between the components of the complex.

Many functions throughout the secretory pathway appear important for metal tolerance since cells defective in vacuolar acidification, endocytosis, exocytosis, vesicular transport, and vacuolar transport displayed enhanced As and Cd sensitivity (Fig 1). The fact that this category was more enriched in the Cd-sensitive gene set indicates that the secretory pathway is more severely affected by Cd than by As. Interestingly, mutants that lack individual components of various protein complexes functioning in protein sorting were metal sensitive; such mutants included components of the HOPS (homotypic fusion and vacuole protein sorting) complex (Vps16p, Vps33p), ESCRT (endosomal sorting complex required for transport) I (Stp22p, Srn2p), ESCRT II (Snf8p, Vps36p, Vps25p), ESCRT III (Snf7p, Vps20p) and GARP (Golgi-associated retrograde protein) complex (Vps51p-52p-53p-54p) (Fig 2A).

The secretory pathway is implicated in many important processes such as lipid biosynthesis, protein targeting and secretion, as well as the unfolded protein response. Consequently, an intact secretory pathway is crucial for the cell to withstand many different environmental conditions. Indeed, mutations impairing proper functioning of the secretory pathway cause sensitivity to a broad range of treatments and growth conditions [41,42]. Metals may impact one or more aspects of the secretory pathway; first, metals may enter the endomembrane system and disturb various processes by interacting with proteins and/or lipids. Secondly, failure to maintain appropriate secretory pathway function may cause metal sensitivity due to defects in delivery or targeting of transmembrane transporters, sensors or signalling receptors. Thirdly, since vacuolar sequestration is important for tolerance acquisition, a decreased capacity to do so is likely to contribute to the observed sensitivity of secretory pathway mutants.

### Arsenite-specific defence functions

Arsenite-specific defence functions that were significantly enriched (*p*-value < 0.005) in our data set were related to cell cycle regulation, lipid and fatty acid metabolism, mitochondrial biogenesis, and the cytoskeleton. The *p*-values and fold enrichment factors are available in Fig 1C and Additional file 1.

#### Cell cycle

Deletion of genes encoding cell cycle related functions produced As-sensitivity; those genes encompassed functions involved in regulation of cell cycle progression (*HOG1*, *PHO85*, *PIN4*, *DBF2*, *CLB2*), as well as spindle body and microtubule formation (*BIK1*, *BIM1*, *CNM67*, *JNM1*, *CIN8*, *CIK1*, *CSM1*, *BNI1*, *CIN1*, *CIN2*, *PAC2*, *TUB3*, *SPC72*). This indicates that cell cycle progression may be targeted by As, confirming our recent finding that As exposure triggers transient G_1 _and G_2 _delays in yeast and that cells defective in Hog1p signalling cannot exit the arsenite-induced G_1 _checkpoint arrest [38]. Similarly, the mechanisms of As action in cancer cells include cell cycle arrest at G_1 _and G_2 _phases, mitotic arrest and subsequent apoptosis. Arsenite can interrupt mitosis by interfering with tubulin polymerization or by disrupting mitotic spindles [12,43]. Elucidating the molecular details of arsenite-induced cell cycle regulation and how this relates to As toxicity and carcinogenicity is important for understanding the potential of As-containing drugs in medical therapy.

#### Mitochondrion

Many mitochondrial proteins are known to be targeted by arsenicals [44]. Indeed, we found several As-sensitive mutants defective in mitochondrial function and biogenesis, including mitochondrial protein biosynthesis and genome maintenance. This observation is consistent with the notion that arsenic toxicity targets mitochondrial processes and that impaired oxidative phosphorylation causes As sensitivity [12,45,46].

#### Lipid and fatty acid metabolism

Deletion of genes encoding functions in lipid and fatty acid metabolism impaired As tolerance; these genes were related to ergosterol (*ERG24, ERG2, ERG3, ERG4, ERG6*), phospholipid (*OPI1, OPI3, ARV1*) and sphingolipid (*DPL1, CSG2, SUR4, DEP1*) biosynthesis. Trace elements and arsenicals appear to affect the fluidity and the thermotropic properties of cell membranes [47,48]. Hence, As may have a direct impact on the lipid bilayer. Alternatively, the lipid composition of cell membranes might be altered in the mutants listed above, thereby changing the properties of the membrane and impacting on the activities of membrane-embedded transporters, sensors or signalling receptors [49]. Interestingly, the activity of the aquaglyceroporin Fps1p, through which arsenite enters cells [36,37] is affected by the plasma membrane ergosterol content [50]. Moreover, an *fps1*Δ mutant has reduced whole-cell and plasma membrane ergosterol levels compared to wild-type cells [50]. Hence, altered plasma membrane ergosterol levels may change Fps1p activity to facilitate arsenite entry.

#### Cytoskeleton

Loss of genes encoding cytoskeletal proteins caused As sensitivity. In particular, mutations affecting the function of the heterohexameric GIM (Genes Involved in Microtuble biogenesis)/prefoldin protein complex, which is required for formation of functional tubulin, sensitized cells to As. These genes include *YKE2 *(*GIM1*), *PAC10 *(*GIM2*), *GIM3*, *GIM4*, *GIM5*, and *PFD1 *(*GIM6*). Analysis of the entire set of As-sensitive mutants for known protein-protein interactions indicated that the proteins encoded by these genes form a highly interacted molecular machine in cells and that disruption of any one of the components of this complex rendered cells sensitive to As (Fig 2B). The cytoskeleton is a known target of As; As triggers actin reorganization in various mammalian cell lines [51,52], and directly interacts with actin as well as with the α and β subunits of tubulin [53,54].

#### Other functions promoting arsenite tolerance

Several other As-specific defence functions were pin-pointed by our analysis (see Additional File 1). Most notable among those is the arsenic resistance gene cluster *ACR3*-*ACR2*-*YAP8 *[55]. *ACR3 *(also called *ARR3*) encodes an arsenite export protein [56], *ACR2 *(*ARR2*) an arsenate reductase [57] and *YAP8 *(*ACR1*/*ARR1*) encodes an arsenic-responsive transcription factor that controls expression of the former two genes [58-60].

### Cadmium-specific defence functions

Cadmium-specific defence functions that were significantly enriched (*p*-value < 0.005) in our data set were mainly related to sugar/carbohydrate metabolism, and metal-ion homeostasis and transport. The *p*-values and fold enrichment factors are available in Fig 1C and Additional file 1.

#### Ion homeostasis and transport

Genes encoding functions in metal-ion homeostasis were enriched in the Cd-specific data set including the ion transporter genes *PCA1 *(Cd exporting ATPase), *TRK1 *(K^+ ^transporter) and *SPF1 *(ER-localized Ca^2+ ^ATPase). Interestingly, the majority of the genes in this category encode functions related to iron homeostasis including the iron regulated transcription factors *AFT2 *and *AFT1*/*RCS1*, the multicopper oxidases *FET3 *and *FET5 *(involved in iron uptake), *FRE6 *(ferric reductase), *FRE8 *(iron/copper reductase) and *ISA2 *(required for maturation of mitochondrial and cytosolic Fe-S proteins). Importantly, while this paper was being written, Ottonello and co-workers reported that iron addition rescues Cd-sensitivity of certain yeast mutants indicating that Cd interferes with iron homeostasis [61]. This is similar to the situation in plants where Cd exposure was shown to provoke iron deficiency [62]. Indeed, expression of several genes related to iron and heme metabolism (*e.g*. *FET3*, *ATX1*, *FRE5*, *ARN1*) is stimulated by Cd [22,32] and during iron deprivation [63,64] providing further support to the notion that Cd-exposed cells experience iron deficiency. Since Fe-S cluster homeostasis regulates transcription of the iron homeostasis systems [65], our data indicate that Cd may affect one or more aspects relating to Fe-S clusters, such as their biosynthesis and/or maturation.

#### Sugar/carbohydrate metabolism

Another Cd-specific defence function appears to involve the glucose sensing Snf1p-pathway since cells lacking *SNF1 *(homologous to mammalian AMP-activated protein kinases), *SNF4*, *REG1, SIP5 *(regulators of Snf1p), and *MIG2 *(transcriptional repressor targeted by Snf1p) were Cd sensitive (see Additional file 1). Snf1p plays a central role in modulating carbon metabolism under glucose limiting and environmental stress conditions. Snf1p also regulates proteins involved in metabolism of reserve carbohydrates such as glycogen and trehalose [66]. Interestingly, we noted that cells lacking either phosphofructokinase (*PFK1 *or *PFK2*) were Cd sensitive. Moreover, deletion of *FBP1*, whose product acts in the reverse step from that catalysed by Pfk1p and Pfk2p also led to Cd sensitivity. Finally, Cd stimulated expression of many genes with functions in glucose metabolism (*GLK1, PDC6, ALD4, VID22, FBP1, FBP26*) [32]. Hence, appropriate functioning of glycolysis may be crucial for Cd tolerance. Cd could impact the cell in such a way that flux through the glycolytic pathway is altered. For example, Cd could have a direct effect on glucose uptake systems [67], on glycolysis itself or on other systems that result in an extra demand on the glycolytic pathway.

### Metal stress tolerance vs. oxidative stress tolerance

**Figure 3:**
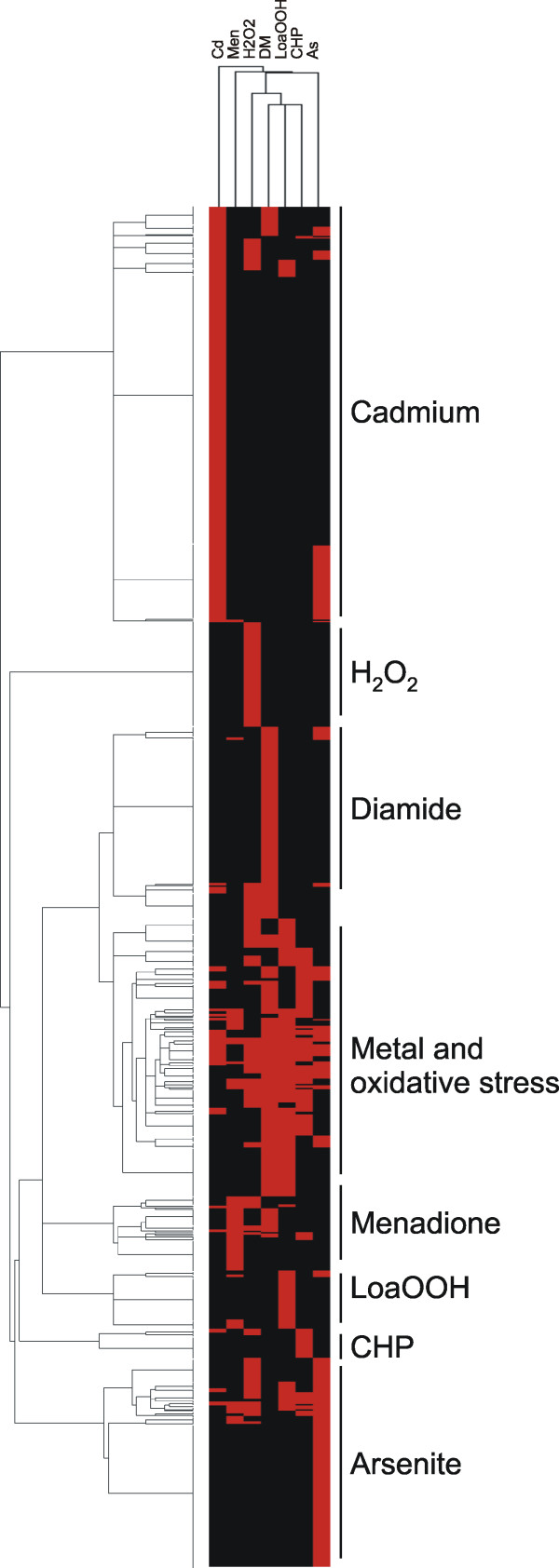
**Genes conferring tolerance to arsenite, cadmium and oxidative stress generating agents**. Hierarchical cluster analysis of mutants that are sensitive to metals or oxidative stress generating agents. Abbreviations used in the heat-map: As (arsenite), Cd (cadmium), H_2_O_2 _(hydrogen peroxide), Men (menadione), CHP (cumene hydroperoxide), DM (diamide), and LoaOOH (linoleic acid 13-hydroperoxide).

The toxicity of As and Cd has in part been attributed to their potential to induce ROS formation and hence to cause oxidative damage in cells [9,68,69]. To address to what extent As, Cd and various oxidants have overlapping toxicity profiles, we compared our gene sets with a set of genes previously reported to mediate tolerance to a number of ROS-generating agents including hydrogen peroxide, menadione, cumene hydroperoxide, diamide, and linoleic acid 13-hydroperoxide [70]. Hierarchical cluster analysis indicated that the genes that confer tolerance to As, Cd and the oxidative stress agents above are to a large extent distinct (Fig 3). Nevertheless, there was a cluster of genes whose absence resulted in sensitivity to metals and oxidative stress. The genes in the metal and oxidative stress cluster were significantly (*p*-value < 0.005) enriched in functions related to mRNA synthesis and transcriptional control, protein synthesis, ribosomal proteins and biogenesis, protein sorting and vacuolar transport, and lipid and fatty acid metabolism. Taken together, some of the genes and cellular functions required for As and Cd tolerance are also necessary for oxidative stress tolerance. However, from the sensitivity profiles it is not possible to draw any conclusions about the source and type of ROS that As and Cd generate and that in turn contribute to their toxicity. Instead, the data indicates that cells employ largely distinct protective mechanisms in different environmental conditions.

### Expression vs. tolerance

In agreement with other studies [71], we found little overlap between genes necessary for As or Cd tolerance and genes whose expression is induced by As or Cd. However, for some defence systems there appears to be such a correlation. For instance, most genes (and enzymes) of the sulphur assimilation and glutathione biosynthesis pathways were strongly up-regulated in response to As and Cd [21,22,32], and many mutants in this pathway were metal sensitive. Another example is genes involved in iron homeostasis: expression of several such genes was induced by Cd whereas their absence resulted in Cd sensitivity. Similarly, expression of the arsenic-resistance genes *ACR2 *and *ACR3 *was stimulated by As and their presence was required for As tolerance (see Additional file 1).

We next asked whether the gene expression profiles of As- and Cd-treated cells would be more similar to each other than the As and Cd sensitivity profiles. As stimulated expression of 334 genes (0.2 mM arsenite, 1 hour) [21] whereas Cd exposure increased expression of 305 genes (0.3 mM Cd, 2 hours) [32] of which 80 genes were common (see Additional file 1). The overlap in those gene sets was about 14% which is similar to the overlap in the sensitive gene sets (18%). Interestingly, the genes induced by both treatments mainly encode functions in sulphur assimilation and glutathione biosynthesis pathways as well as in (oxidative) stress responses and detoxification, and many are transcriptionally regulated by Met4p and Yap1p. Hence, several processes that contribute to both As and Cd tolerance are transcriptionally stimulated by these metals. We also checked the overlap between the common As/Cd sensitivity gene set (106 genes) and the common As/Cd-induced gene set (80 genes). Curiously, these gene sets only had three genes in common (*MET16, MET17, CYS3*). Hence, although the two gene sets (phenotype and expression) are enriched for genes encoding related functions, the identities of those genes are largely distinct.

### Human orthologues of yeast genes mediating arsenite and cadmium tolerance

We next asked to what extent the genes in our As- and Cd-sensitive gene set have human homologues. Using the HomoloGene database [72] we found that 43 of the 106 genes in the common As/Cd sensitive gene set have at least one human homologue (41%) (see Additional file 1). These proteins are involved in diverse functions in humans including signalling, transcription, chromatin modification, and vacuolar protein sorting. Moreover, these genes were significantly (*p*-value < 0.005) enriched for functions related to carbohydrate metabolism as well as protein targeting and sorting. We conclude that similar biological processes may act in yeast and humans to prevent metal toxicity and carcinogenesis.

### Comparison of this study to other genome-wide screens

**Figure 4:**
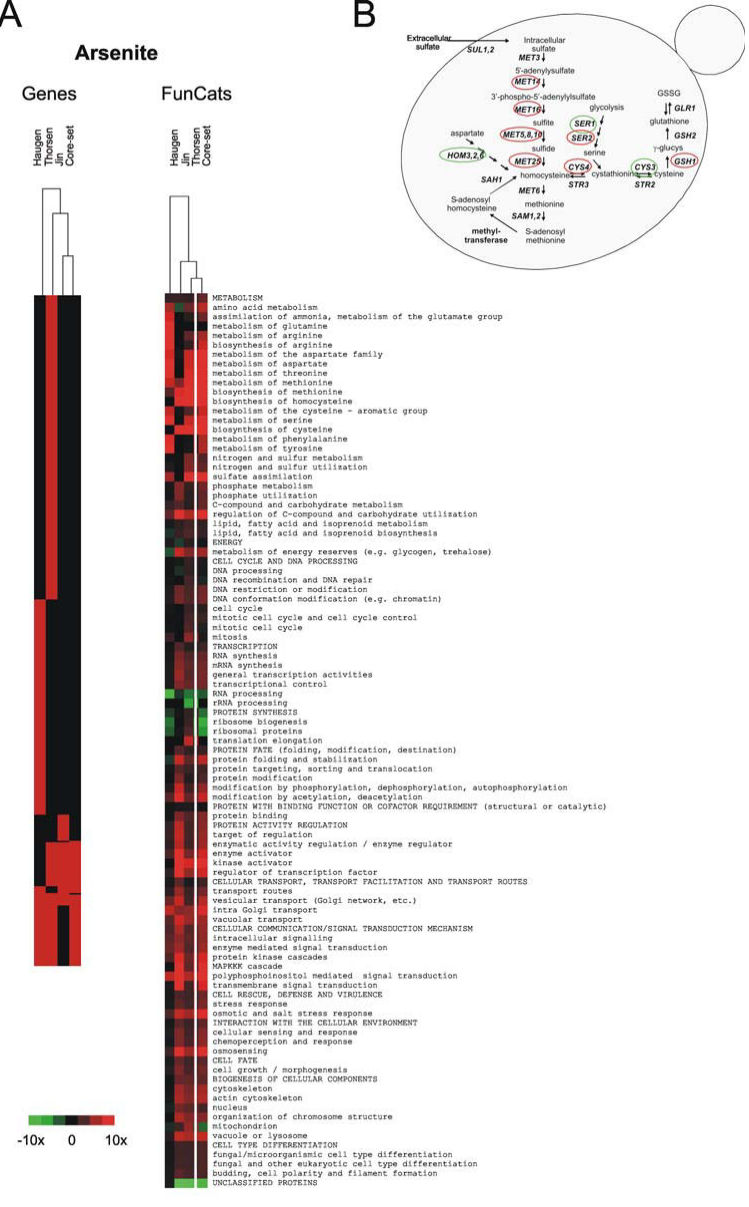
**Comparative analysis of arsenite sensitivity data**. (**A**) Comparison and visualization of arsenite sensitivity data from this work and other studies [31,33]. Hierarchical cluster analysis of mutants that were scored as As sensitive and of the functional categories (according to FunCats (MIPS)) in the corresponding data sets that were significantly (*p*-value < 0.005) enriched for As sensitive mutants in at least one of the studies. Parent terms are in capitals, child terms in lower case letters. The colour indicates the fold enrichment of genes within individual categories. (**B**) Arsenite sensitive mutants in the sulphur assimilation and glutathione biosynthesis pathways identified by individual screens (this work and [31,33]). Red circles: identified by one screen only; green circles: identified by at least two studies.

**Figure 5:**
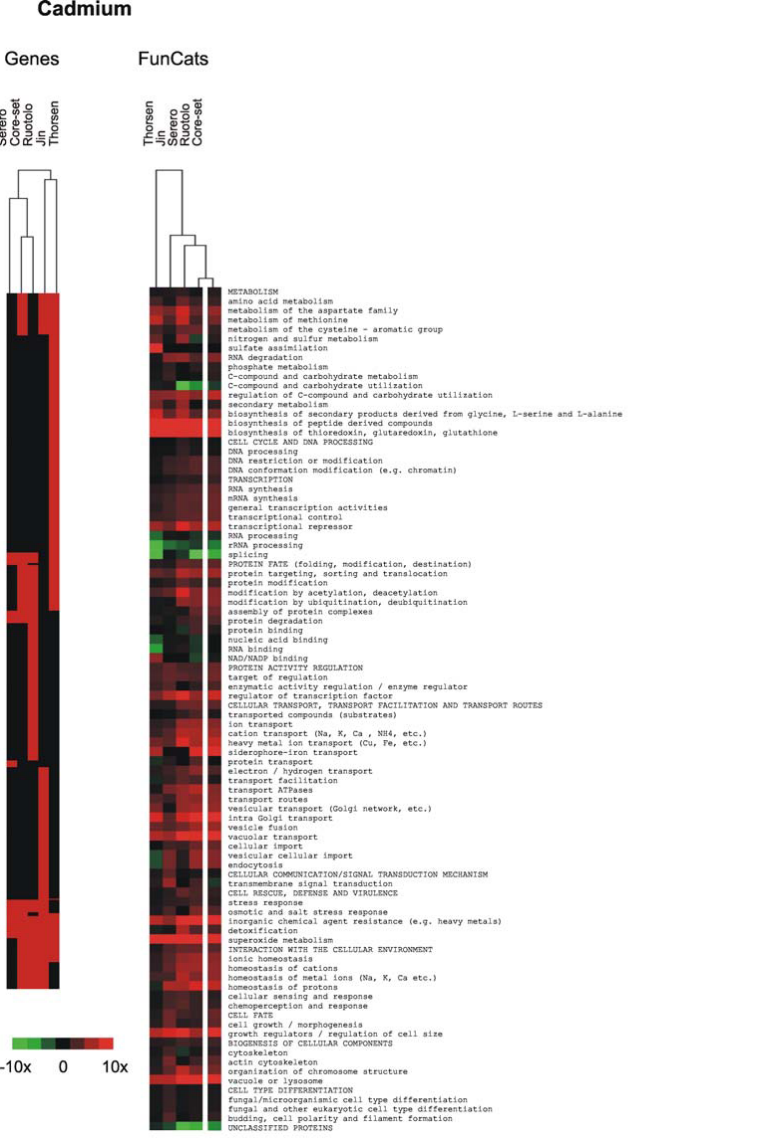
**Comparative analysis of cadmium sensitivity data**. Comparison and visualization of cadmium sensitivity data from this work and other studies [33,61,74]. Hierarchical cluster analysis of mutants that were scored as Cd sensitive and of the functional categories (according to FunCats (MIPS)) in the corresponding data sets that were significantly (*p*-value < 0.005) enriched for Cd sensitive mutants in at least one of the studies. Parent terms are in capitals, child terms in lower case letters. The colour indicates the fold enrichment of genes within individual categories.

While preparing this manuscript, several genome-wide phenotypic screens were published that included arsenite and cadmium sensitivity data [31,33,61,73,74]. Haugen and co-workers [31] found 214 As-sensitive mutants of which 53 (11%) were present also in our data set, whilst Jin *et al *[33] identified 65 As-sensitive mutants, 40 (12%) of which were common to our study. 303 Cd-sensitive mutants were found by Ruotolo and colleagues [61], 73 by Serero and co-workers [74], and 276 by Jin *et al *[33]. Of those, 106 (18%), 39 (9%) and 94 (17%) respectively were present in our gene set whereas the overlap between the Ruotolo and Serero gene sets was 19%. Hence, the overlap in terms of mutants is roughly 10–20% and this relatively poor overlap is clearly visible in the heat maps in Figures 4 and 5. A reason for this discrepancy could be that none of the screens is saturated. Moreover, the corresponding screens were performed in dissimilar conditions e.g. on solid vs. liquid medium or screening mutants individually vs. in a mixture using a TAG array approach. Another factor likely to affect the outcome of a screen is the way sensitive genes are identified e.g. by visual inspection, TAG array hybridization, continuous measurements of optical density or by scoring growth after a specific time has elapsed. These factors probably influence the number and identity of mutants that are scored to affect growth. Despite the discrepancy, we reasoned that the gene sets probably contain relevant biological information. We therefore looked for mutants that were consistently identified as sensitive in at least two of the studies; these core-sets contained 89 As-sensitive and 209 Cd-sensitive mutants (see Additional file 1). The As core-set was enriched in functions related to sulphur metabolism, environmental sensing and signalling, transcription, and the cytoskeleton whereas the Cd core-set was enriched in functions related to sulphur and glutathione biosynthesis, transcription, intracellular transport and sorting, regulation of carbon metabolism, and metal-ion homeostasis and transport (Figs 4 and 5). Overall, these categories were similar to those found by us and others (Fig 1; [31,33,61,74]). As-defence genes identified in all studies included *YAP8*, *ACR3 *and *YCF1 *whilst Cd-defence genes found in all studies included *GSH1*, *GSH2*, *YCF1*, *YAP1 *(glutathione biosynthesis), *BCK1*, *SLT2 *(MAP kinase signalling), as well as genes related to transcription and intracellular transport. Importantly, hierarchical cluster analysis of functional categories that were significantly (*p*-value < 0.005) enriched for metal sensitive mutants revealed a much better correlation than on the gene level (Figs 4 and 5). Hence, although these screens identified largely non-overlapping gene sets, they did uncover similar biological functions. In several cases, the studies above identified different mutants encoding proteins in the same pathway. One such example is the sulphur assimilation and glutathione biosynthesis pathways in the As-sensitive gene sets; 5 genes were common to at least two of the screens whereas 9 were found by only one study (Fig 4B). Of those, 14 were identified by us, four by Haugen and coworkers [31] and one by Jin *et al *[33]. Similarly, all six components of the GIM complex were found by us, four by Jin *et al *[33] and none by Haugen and co-workers [31]. Taken together, since the functional categories affected by metals found in each study clustered well, we conclude that genome-wide phenotypic screens provide biologically relevant insight into cellular systems mediating tolerance. However, a single screen is unlikely to uncover all genes and systems conferring tolerance.

### Molecular clues to arsenite and cadmium toxicity and tolerance mechanisms

Next, we wanted to exploit the information in our data sets to discover toxicity and tolerance mechanisms at a molecular level. To do so, we explored the role of two cellular systems identified above in more detail, one related to arsenite and one related to cadmium.

#### The cytoskeleton is targeted by arsenite

**Figure 6:**
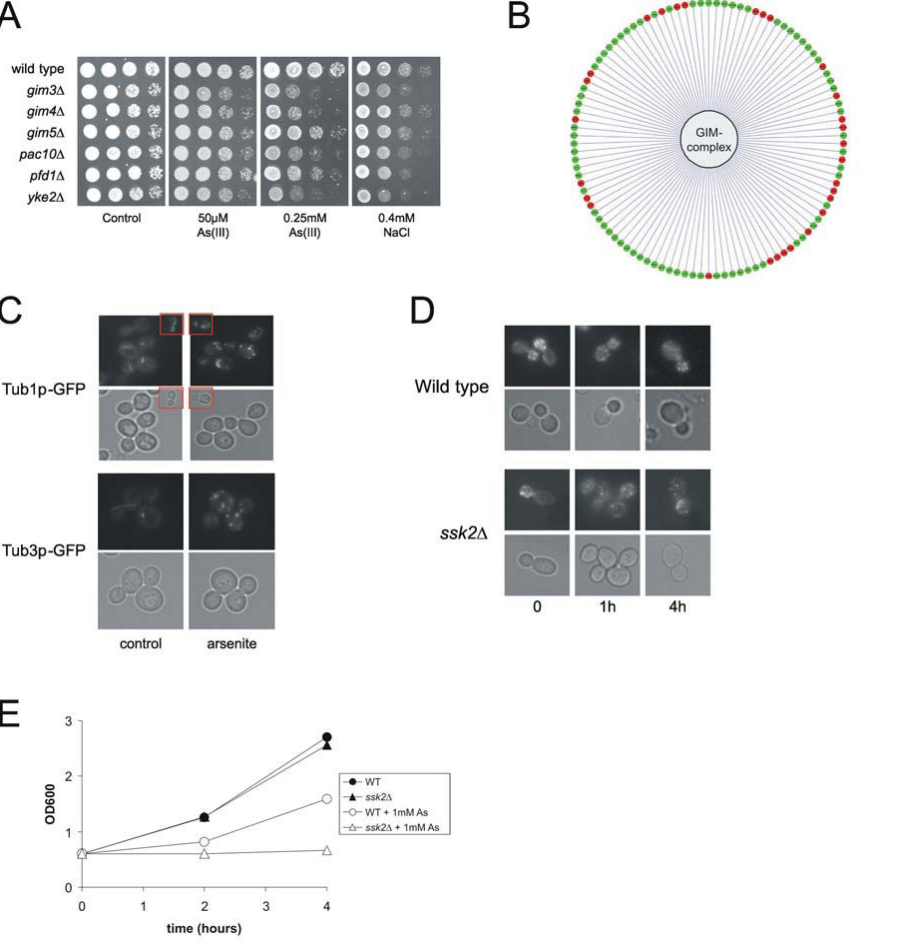
**Arsenite targets the cytoskeleton**. (**A**) The GIM mutants are strongly arsenite sensitive. 10-fold serial dilutions of exponentially growing cells were spotted on YEPD agar plates with arsenite or NaCl. Growth was scored after 2–3 days at 30°C. (**B**) Synthetic lethal (SL) interactions of the GIM complex. Those genes that show SL interactions with at least four of the GIM genes are shown and the red colour indicates arsenite sensitivity. (**C**) Morphology of the microtubule cytoskeleton. Chromosomally encoded Tub1p-GFP and Tub3p-GFP were visualized in living cells before (control) and 1 hour after exposure to 1 mM arsenite. (**D**) Morphology of the actin cytoskeleton. Actin was stained with rhodamine-phalloidin in wild-type and *ssk2*Δ cells before and after exposure to 1 mM arsenite. (**E**) Growth of wild-type and *ssk2*Δ cells in the presence of 1 mM arsenite.

Loss of any component of the heterohexameric GIM/prefoldin complex sensitized cells to As. The GIM complex is involved in actin and tubulin folding, and GIM mutants are defective in actin and tubulin organization, and are cold- and osmosensitive [75-77]. Interestingly, whereas GIM mutants are sensitive only at relatively high osmolarity, they are sensitive already at very low As concentrations; these mutants grew poorly at 50 μM arsenite as evident from the small colonies formed (Fig 6A). To gain further insight into the role of the GIM complex, we analysed all synthetic lethal (SL) interactions between GIM genes and the genome (see Additional file 1). Of the 107 genes showing SL interactions with at least four of the GIM genes, 29 were also As-sensitive (Fig 6B) indicating a significant enrichment (Fischer's test: 9.25 E^-10^) in As-sensitivity among GIM-interacting genes. These 29 genes encoded cytoskeletal proteins and functions that involve the cytoskeleton such as the cell cycle, budding and cell polarity. This data can be interpreted in two ways; As might target the GIM complex directly and that is the reason why the GIM mutants are sensitive. Alternatively, As might target the SL interacting gene-products and the cells cannot cope with defects in the GIM complex at the same time. Given that most of the interacting genes perform functions that require a functional GIM complex argues that the complex itself is directly targeted by As.

To further explore how As affects the cytoskeleton, we monitored α-tubulin organization by following GFP-tagged Tub1p and Tub3p. In the absence of As, extended fluorescence arrays were visible in most cells. In contrast, the microtubule arrays decreased dramatically in response to As exposure indicating disassembly of cytoplasmic microtubules (Fig 6C). Also the actin cytoskeleton was strongly affected by As. In untreated wild-type cells, actin patches were predominantly localized to the buds whereas As exposure triggered a temporary depolarization of the actin cytoskeleton with an even distribution of patches in the bud and the mother cell (Fig 6D). After 4 hours, the actin cytoskeleton recovered its polar distribution in nearly all cells and this recovery coincided with growth resumption (Fig 6E). Ssk2p is a kinase that activates the MAP kinase Hog1p in response to osmotic stress. In addition, Ssk2p binds to actin and promotes actin cytoskeleton recovery after osmotic stress [78]. We previously demonstrated that *ssk2*Δ cells are As sensitive and that this sensitivity is unrelated to Hog1p phosphorylation [36]. Instead, Ssk2p might have a role in actin recovery also during As-exposure; actin remained depolarized in As(III)-treated *ssk2*Δ cells (Fig 6D) and the mutant could not resume growth (Fig 6E). Taken together, these results provide strong support for the cytoskeleton being a direct target of As toxicity and that As inhibits the activity of the GIM complex *in vivo*. Hence, arsenite has a dual effect on the cytoskeleton; it binds to and disrupts the actin and tubulin cytoskeleton, and it inhibits the GIM complex that is required for folding of *de novo *synthesised actin and tubulin monomers.

#### Snf1p phosphorylation is required for Cd tolerance

**Figure 7:**
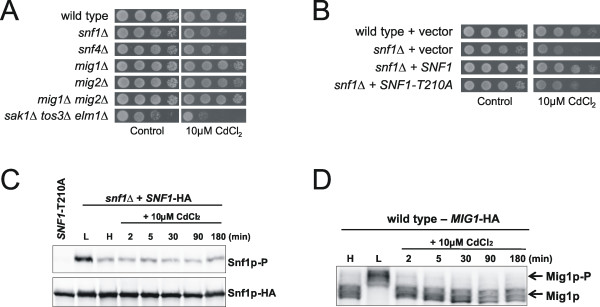
**Snf1p phosphorylation is required for Cd tolerance**. (**A**) Cells lacking Snf1p or proteins regulating its activity (Snf4p or the upstream kinases Sak1p, Tos3p, Elm1p) are cadmium sensitive. 10-fold serial dilutions of exponentially growing cells were spotted on YNB + 2% glucose agar plates containing cadmium and growth was scored after 2–3 days at 30°C. (**B**) Snf1p phosphorylation/kinase activity is required for cadmium tolerance.*snf1*Δ cells were transformed with a plasmid containing *SNF1 *or the unphosphorylatable *SNF1*-T210A allele. Wild-type and *snf1*Δ cells were also transformed with the empty vector as a control. 10-fold serial dilutions of exponentially growing cells were spotted on selective medium with or without cadmium, and growth was scored after 2–3 days at 30°C. (**C**) Snf1p phosphorylation is not induced by cadmium. Snf1p phosphorylation was monitored with a phospho-specific-Snf1p antibody whereas total Snf1p was detected with anti-HA antibody. H: high glucose (2%); L: low glucose (0.05%). (**D**) Mig1p is not phosphorylated during cadmium exposure. Mig1p was detected with anti-HA antibody and mobility was monitored in the presence of cadmium, low glucose (L: 0.05%) or high glucose (H: 2%).

The phenotypic data from our screen indicated a role of the glucose sensing Snf1p pathway for Cd tolerance. The Snf1p kinase is a heterotrimeric complex consisting of the catalytic α-subunit Snf1p, the regulatory γ-subunit Snf4p, and one of the three β-subunits (Sip1p, Sip2p, Gal83p). Snf1p activation involves Snf4p and any of the redundant upstream kinases Sak1p, Tos3p and Elm1p that phosphorylate Snf1p on threonine 210 (T210) [66]. We found that *snf1*Δ, *snf4*Δ and the *sak1*Δ *tos3*Δ *elm1*Δ triple mutant were Cd sensitive (Fig 7A) suggesting that Snf1p phosphorylation is important for Cd tolerance. Indeed, the unphosphorylatable *SNF1*-T210A allele was unable to rescue growth of *snf1*Δ in the presence of Cd (Fig 7B). To monitor Snf1p phosphorylation directly, we treated cells with cadmium (or low glucose as a control), prepared cell extracts and performed Western blot analysis using an anti-phospho-Snf1p antibody (Fig 7C). Basal Snf1p phosphorylation did not increase upon Cd-treatment whereas low glucose triggered strong Snf1p phosphorylation. No phosphorylation was detected in cells expressing the *SNF1*-T210A allele. We also monitored phosphorylation of the transcriptional repressor Mig1p, which is targeted by Snf1p under low glucose conditions. Cd did not trigger Mig1p phosphorylation whilst Mig1p was strongly phosphorylated in low glucose conditions (Fig 7D). Together with the fact that *mig1*Δ is not Cd sensitive (Fig 6A), these results indicate that Snf1p action in Cd tolerance is different from that under low glucose conditions. Nevertheless, our data clearly shows that a low level of Snf1p kinase activity is necessary and sufficient to confer significant Cd tolerance to yeast cells. In line with this observation, low level of Snf1p kinase activity is sufficient to confer tolerance to other toxic agents like lithium and hydroxyurea [79,80] although the mechanisms are not well understood. To conclude, high level of Snf1p kinase activity is required for growth in the absence of glucose while a low level of kinase activity is sufficient to confer tolerance to toxic agents when glucose is present.

## Conclusion

This study has highlighted the importance of individual genes, pathways and cellular functions that protect yeast cells against arsenite and cadmium toxicity. We have pinpointed common as well as metal-specific defence functions and provided novel insight into As and Cd tolerance systems and toxicity targets. In particular, molecular evidence indicates that the cytoskeleton is a direct target of arsenite toxicity, and that the GIM complex is affected by arsenite *in vivo*. We also demonstrate that phosphorylation of the Snf1p kinase is necessary for Cd tolerance. These aspects of arsenite/cadmium toxicity and tolerance are novel and add to our understanding of metal action and tolerance acquisition mechanisms.

The fact that As and Cd has been included in some recent genome-wide phenotyping screens [31,33,61,73,74] enabled us to assess the overlap between and the reliability of these studies. Although the overlap on the gene level was rather poor, the corresponding studies identified similar biological processes that are important for tolerance acquisition. The discrepancy at the gene level might be due to variations in experimental settings and/or due to that the screens are not saturated. Nevertheless, genome-wide phenotypic screens provide biologically relevant insight into tolerance mechanisms, and phenotypic profiling under different conditions may enhance the reliability of the data.

What can we conclude about the mode of action of metals based on a genome-wide sensitivity screen? Even though phenotypic profiling pin-points core functions that confer metal tolerance, one cannot firmly conclude that those gene-products represent direct toxicity targets. In fact, many phenotypes could be a result of synthetic effects, e.g. the lacking gene product and the metal may affect parallel pathways/protective systems. A complementary approach may involve a search for genes that confer resistance upon overexpression since a gene that confers resistance when overexpressed and sensitivity when absent is more likely to be a direct target of the metal. However, it is clear that molecular and biochemical studies will ultimately be required to confirm whether a protein is indeed targeted by a metal or not. In this study, we provide both genomic and molecular evidence that the cytoskeleton and the GIM/prefoldin complex are targets of arsenite toxicity.

To conclude, this study has shed light on the genetic basis of arsenite and cadmium tolerance in *S. cerevisiae*. This catalogue of genes and protective functions will be instrumental for generating hypotheses about the role of individual factors for tolerance acquisition and for providing insights into the modes of metal action. Unveiling metal toxicity and tolerance mechanisms in yeast may prove of value for identifying similar mechanisms in higher eukaryotes.

## Methods

### Strains, plasmids, growth conditions and reagents

The complete set of viable yeast mutants in the haploid strain BY4741 (*MAT**a **his3*Δ*1 leu2*Δ*0 met15*Δ*0 ura3*Δ*0*) and the homozygous diploid strain BY4743 (*MAT**a**/MAT**α **his3*Δ*1/his3*Δ*1 leu2*Δ*0/leu2*Δ*0 ura3*Δ*0/ura3*Δ*0 LYS2/lys2*Δ*0 met15*Δ*0/MET15*) from EUROSCARF [81] were screened for growth in the presence of various concentrations of cadmium chloride (Sigma) and sodium arsenite (Sigma). The strains were grown in the absence or presence of metal on YEPD (1% yeast extract, 1% peptone, 2% glucose) or YNB (0.67% yeast nitrogen base) medium supplemented with complete amino acid supplement mix (BIO101) and 2% glucose as carbon source. Tub1p-GFP and Tub3-GFP strains were obtained from Invitrogen, and the Snf1p pathway mutants (in W303-1A strain background) and the centromeric plasmids containing HA-tagged *SNF1 *and *SNF1*-T210A are described in [82]. The *MIG1 *gene was epitope tagged by chromosomal integration of a PCR amplified triple HA-tag using primers 5'-ACA AAC CCC CAT TTC TCA GTC GGA TTC ACA AGT TCA AGA ACT GGA AAC ATT ACC ACC CAT AAG AAG TTT ACC GTT GCC CTT CCC ACA CAT GGA CCG TAC GCT GCA GGT CGA C and 5'-ATT ATT TAT TAT TTA TTA ATT ATT AAT TGT TAA TAT TAT TAA TTC TTG TCT ATT GTC TTT TGA TTT ATC TGC ACC GCC AAA AAC TTG TCA GCG TAT CAA TGC TCG TTA AAG TGT GTG GTT, and plasmid pYM22 [83].

### Screening and scoring for metal sensitivity

An ordered array of ~4700 viable gene deletion mutants in the haploid BY4741 background was screened essentially as described in [84]. Strains were replica pinned onto YNB control plates and plates with cadmium chloride (75, 100 and 150 μM) or sodium arsenite (0.5, 1.0 and 1.5 mM) using a 96-floating pin replicator operated by a Biomek 2000 robot (Beckman). The strains were arrayed in quadruplet to create a dilution in a given square giving a total of 96 strains plated per agar plate (see Additional file 2). The plates were incubated at 30°C and photographed after 24, 48 and 72 hours. The images were quantified in the image analysis software ImaGene v.6 (Biodiscovery) and spot area and intensity were extracted. The resulting data was then imported and analyzed in the statistical language R [85]. To remove technical artefacts and non-growing colonies the spots were filtered based on spot area and intensity. First, all control spots with an area less than 16 were removed. Then, spots with an area greater or equal to 16 but with the intensity within the spot less than the background intensity minus one standard deviation were: (1) removed if it was a control spot or (2) area set to 1 if it was a mutant. The logarithm of the fold-change between the mutant and control area was used as an estimate of the effect of metals on growth, where resistance and sensitivity is indicated by positive and negative values respectively. The logarithmic fold-changes were then normalized by subtracting an estimate plate effect, which was based on calculating the logarithmic fold-change between the median of the spots on the plates in question. Finally, the four replicates were added together using a trimmed mean value, where the highest and the lowest values were removed. Mutants with an average fold-change greater than 1.5 were selected for subsequent analysis.

The homozygous diploid BY4743 collection was screened essentially as described in [73]. In brief, cells were grown at 30°C in liquid YEPD medium with or without metal and cell density of each culture (OD_600_) was measured after 24 and 48 hours. Sensitive mutants were re-screened to confirm slow growth in the presence of metal. Finally, the sensitive gene-sets from both screens (haploid and homozygous diploid) were pooled and used for further analysis.

### Data analyses

To pin-point cellular functions that confer metal tolerance, we searched for functional categories (according to FunCat, Munich Information Center for Protein Sequences (MIPS)) [20] that were significantly enriched (*p*-value < 0.005) in the sensitive gene-set as compared to the whole genome. Gene Ontology (GO) analysis was done using GO Term Finder [86], putative human homologues of As and Cd sensitive yeast genes were identified using the National Center for Biotechnology Information HomoloGene database [72], and affinity MS data for protein-protein interactions was obtained from [86]. Hierarchical clustering of metal and oxidative stress sensitive data was performed using Cluster 2.11 and visualized with Java TreeView 1.1.3 [87].

### Immunodetection, microscopy and staining methods

Exponentially growing cells were either untreated or exposed to Cd, harvested and disrupted as described previously [82]. Proteins were separated by SDS-PAGE and analysed using anti-phospho-Snf1p antiserum (Open Biosystems) or anti-HA antibody (Santa Cruz Biotechnology). IRDye goat 800CW anti-mouse or IRDye 680 anti-rabbit IgG were used as secondary antibodies. Filters were incubated with Odyssey blocking buffer (LI-COR Biosciences) and visualised using Odyssey IR scanner (LI-COR Biosciences). To monitor Tub1p-GFP and Tub3p-GFP, cells were grown in YEPD medium to mid-log phase, washed twice with phosphate-buffered saline (PBS) and the GFP signals were observed in living cells before and after exposure to arsenite. To visualize actin, cells were untreated or exposed to arsenite, and incubated with rhodamine-conjugated phalloidin (Invitrogen) as described in [88]. The GFP and rhodamine signals were observed using a Leica DM R fluorescence microscope.

## Abbreviations used

ABC: ATP-binding cassette; MAP: mitogen-activated protein; ROS: reactive oxygen species; HOG: high osmolarity glycerol; As: arsenic; Cd: cadmium; Fe-S: iron-sulphur; SL: synthetic lethal; GFP: green fluorescent protein; PBS: phosphate-buffered saline.

## Authors' contributions

MJT, GGP and MT conceived and designed the study; MT and GGP performed the genome-wide screens; TY performed phosphorylation experiments; MT, EK, GGP, MT and MJT analyzed the data; MJT, GP, EK, MT, IWD and ON participated in writing the paper; all authors approved the final manuscript.

## Supplementary Material

Additional file 1 **Gene lists and data used for analysis. **Excel sheet containing all gene lists and data used for analysis including As- and Cd-sensitive gene-sets, GO-annotations, FunCat analysis, and human homologues of yeast genes. Click here for file

Additional file 2 **Representative 384-well plate from the primary metal screen.** Figure showing a representative 384-well plate from the primary metal screen. Click here for file
