# Supplementary material for: Genetic basis of arsenite and cadmium tolerance in Saccharomyces cerevisiae
Source: BMC Genomics. 2009 Mar 12;10:105. doi: 10.1186/1471-2164-10-105 (PMC2660369; doi:10.1186/1471-2164-10-105)
Supplement: Additional file 2 — Representative 384-well plate from the primary metal screen. Figure showing a representative 384-well plate from the primary metal screen. [file 1471-2164-10-105-S2.pdf]

A

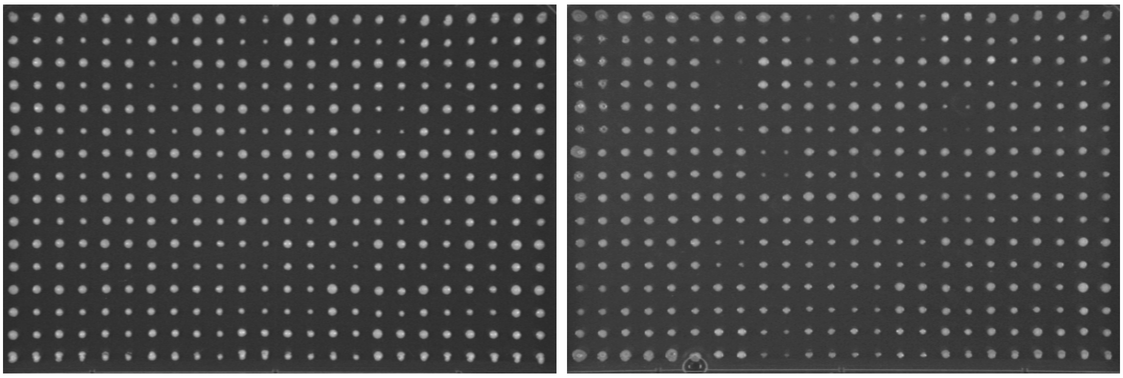

Control 1.5 mM As(III)

B

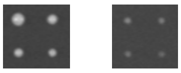

Control 1.5 mM As(III)

Representative 384-well plate from the primary metal screen. A set of 96 yeast mutants is shown (A), each pinned four times to create a dilution (B) onto solid medium without and with 1.5 mM arsenite. Small colonies represent putative arsenite-sensitive mutants. Plates were photographed after 48h at 30°C.

Additional File 2: Thorsen et al
